# Primary Cutaneous Peripheral T-Cell Lymphoma Not Otherwise Specified: A Rapidly Progressive Variant of Cutaneous T-Cell Lymphoma

**DOI:** 10.1155/2015/429068

**Published:** 2015-08-26

**Authors:** Kimberly Aderhold, Lisa Carpenter, Krysta Brown, Anthony Donato

**Affiliations:** Department of Internal Medicine, Reading Health System, West Reading, PA 19608, USA

## Abstract

Primary Cutaneous Peripheral T-Cell Lymphoma NOS (PTL-NOS) is a rare, progressive, fatal dermatologic disease that presents with features similar to many common benign plaque-like skin conditions, making recognition of its distinguishing features critical for early diagnosis and treatment (Bolognia et al., 2008). A 78-year-old woman presented to ambulatory care with a single 5 cm nodule on her shoulder that had developed rapidly over 1-2 weeks. Examination was suspicious for malignancy and a biopsy was performed. Biopsy results demonstrated CD4 positivity, consistent with Mycosis Fungoides with coexpression of CD5, CD47, and CD7. Within three months her cancer had progressed into diffuse lesions spanning her entire body. As rapid progression is usually uncharacteristic of Mycosis Fungoides, her diagnosis was amended to PTL-NOS. Cutaneous T-Cell Lymphoma (CTCL) should be suspected in patients with patches, plaques, erythroderma, or papules that persist or multiply despite conservative treatment. Singular biopsies are often nondiagnostic, requiring a high degree of suspicion if there is deviation from the anticipated clinical course. Multiple biopsies are often necessary to make the diagnosis. Physicians caring for patients with rapidly progressive, nonspecific dermatoses with features described above should keep more uncommon forms of CTCL in mind and refer for early biopsy.

## 1. Introduction

Primary Cutaneous Peripheral T-Cell Lymphoma not otherwise specified (PTL-NOS) is an aggressive and life threatening dermatologic malignancy that often presents mimicking other less severe plaque-like skin conditions. Due to the nonspecific nature of these lesions, CD4-positive CTCL is often misdiagnosed as either Mycosis Fungoides or Sezary Syndrome. It is not until the disease progresses that further investigation into alternative diagnoses is prompted. Biopsy and immunohistochemical analysis of CTCL are nonspecific and a discrepancy in the clinical picture may require the diagnosis to be amended. The 5-year survival rate for PTL-NOS is less than 20%, which makes timely diagnosis an important measure to improve outcome [1]. We describe a patient who presented with a single malignant nodule which rapidly progressed provoking diagnosis with the rarest form of CTCL, known as PTL-NOS.

## 2. Case

A 78-year-old female without significant past medical history presented to an outpatient surgery center with a two-week history of a rapidly growing nodule on her right shoulder. The patient noted that the nodule spontaneously bled as it became caught on clothing but otherwise was asymptomatic. She applied Polysporin topically without signs of healing or regression of the lesion. There were no other lesions of concern. She admitted to malaise but denied weight loss, night sweats, fevers, or loss of appetite. Her family history was negative for any malignancies.

**Figure 1:**
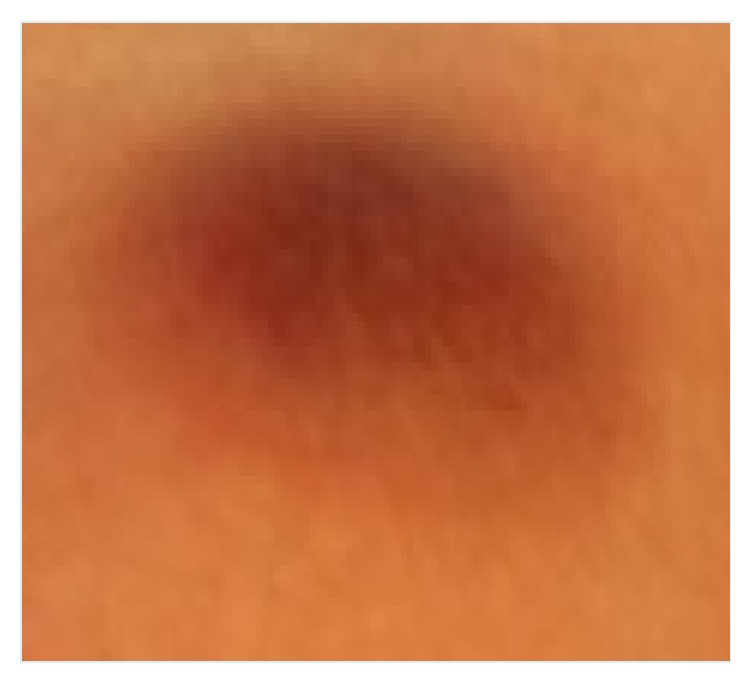
Initial presentation of the 5 cm nodule found on the right posterior shoulder.

On examination, the patient had a solitary 5 cm ill-defined, red-violaceous, immobile, irregular nodule on her right posterior shoulder (Figure 1). The lesion was not indurated or fluctuant and was nontender to palpation. There was a small area of excoriation with crusting. Due to the malignant features of the lesion, a 3 mm punch biopsy was performed. Laboratory or imaging tests were not performed at the time of biopsy.

Biopsy revealed a dense pan-dermal infiltrate of medium- to large-sized atypical lymphocytes with irregular nuclei and perinuclear halos (>25% large cells). The lymphocytes exhibited epidermotropism, blurring the dermal-epidermal junction and epidermis. The infiltrate formed large groups within the epidermis and extensively infiltrated the subcutaneous adipose tissue. Scattered mitotic figures were also identified. Immunohistochemical staining showed CD4-positive T-cell population that coexpressed CD5, CD45, and CD7. EBV, CD30, and CD56 were negative.

**Figure 2:**
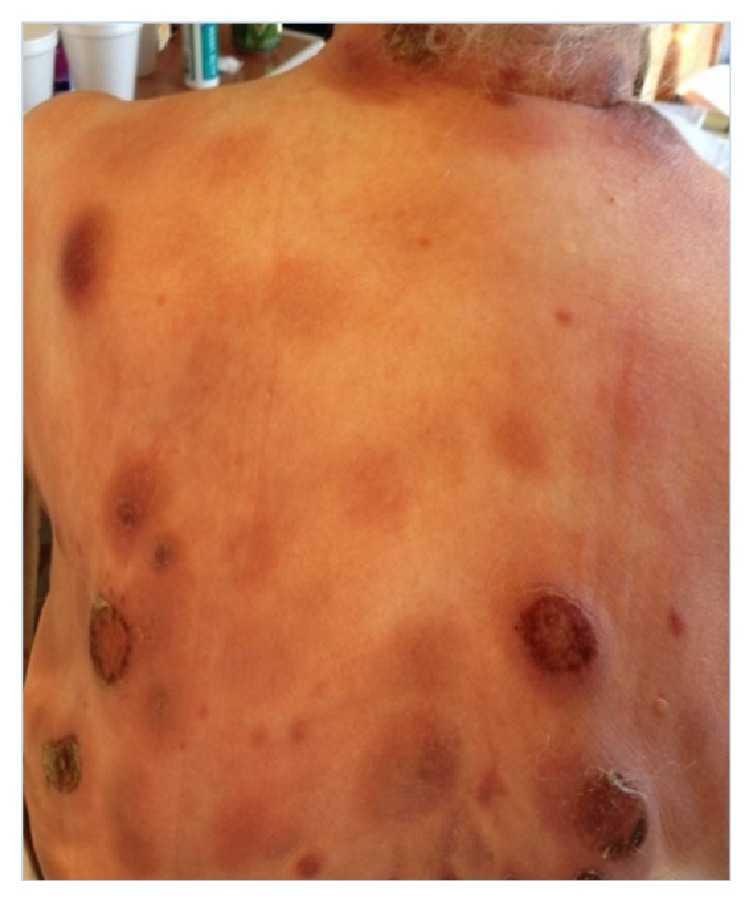
Diffuse, multifocal red-violaceous nodules on the patient's back and neck. Several nodules are ulcerated and crusted and are exudative.

**Figure 3:**
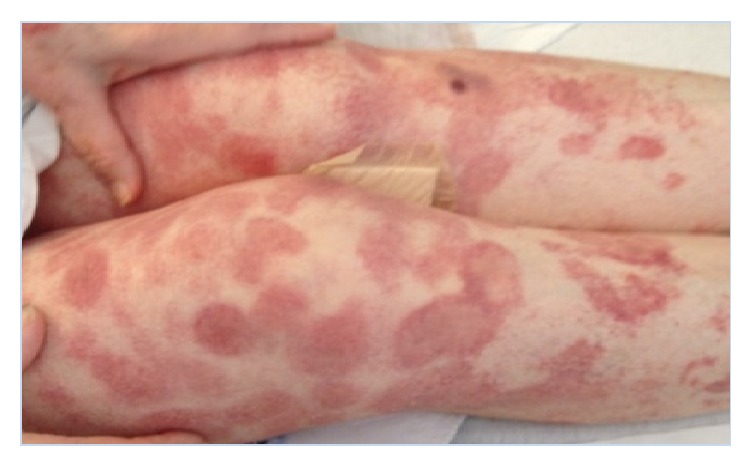
Multiple scattered irregular nodules on the patient's legs. Bandage over excoriated lesion.

**Figure 4:**
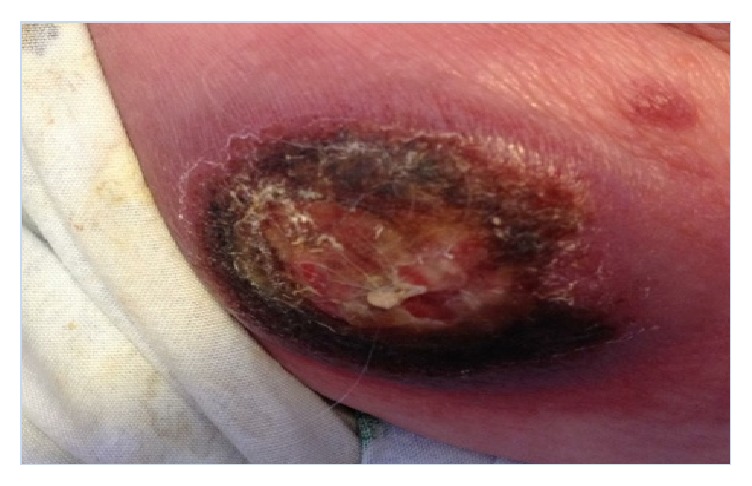
Ulcerated, necrotic, exudative nodule found on patient's R lateral thigh.

These results prompted surgical excision with wide margins and referral to a hematology/oncology specialist. The patient was initially diagnosed with Mycosis Fungoides and was started on Methotrexate. Unfortunately, the Methotrexate proved to be ineffective, and over the next three months her disease continued to progress with multiple new nodules and plaques arising over several areas of her body (Figures 2 and 3). Several nodules became ulcerated and productive of purulent fluid (Figure 4). She additionally developed a lesion suspicious for metastasis in her spleen that was seen on CT scan. This lesion was not biopsied per the patient's request.

After recognizing the aggressive nature of her disease, her diagnosis was amended to Primary Cutaneous Peripheral T-Cell Lymphoma, NOS. She was started on combination Cyclophosphamide, Hydroxydaunomycin (Doxorubicin), Oncovin (Vincristine), and Prednisone. The disease partially responded to chemotherapy with regression of lesions on her lower extremities. Unfortunately the chemotherapy was poorly tolerated by the patient and, after two cycles of chemotherapy, she declined any further treatment. She eventually succumbed to sepsis due to skin barrier breakdown six months after diagnosis.

## 3. Discussion

Cutaneous T-Cell Lymphoma (CTCL) is a form of Non-Hodgkin Lymphoma that involves neoplasms of T-cells primarily concentrating within the skin [2]. CTCL can vary considerably in clinical presentation, prognosis, and histological and immunophenotypical description [2]. The most common forms of CTCL are Mycosis Fungoides and Sezary Syndrome, which account for about 65% of all CTCL cases [2]. However, Primary Cutaneous Peripheral T-Cell Lymphoma not otherwise specified (PTL-NOS) is the rarest form of CTCL and is a diagnosis of exclusion based on criteria from the World Health Organization (see below) [1, 2].

Patients with PTL-NOS may present with a solitary red-violaceous tumor-like nodule on any area of the body; however, most commonly patients present with scattered multifocal or diffuse nodules [2]. Many of these tumors become ulcerated and subsequently infected. Rapid dissemination of the cutaneous tumors and systemic involvement are unfortunate key features of PTL-NOS contributing to the five-year survival rate of less than 20% [1, 2].

Due to the rarity of cases, PTL-NOS is not well understood and algorithms for treatment are lacking. PTL-NOS falls within the rarest CTCL subtype of Primary Cutaneous Lymphomas (PCLs) and accounts for only about 2% of PCLs [1, 2]. PTL-NOS may be difficult to diagnose due to the variability of its immunophenotypes; most commonly tumors are CD4-positive with a variable loss of almost all T-cell antigens [2]. The CD30 phenotype is usually limited or absent and rarely CD56 may stain positive [2]. Although epidermotropism is generally mild or absent, histopathologically PTL-NOS will display nodular or diffuse infiltrates of medium- to large-sized pleomorphic or immunoblastic T-cells [2].

The WHO-EORTC established a set of criteria to help define PTL-NOS from other, more well-defined rare subtypes of Primary Cutaneous Lymphomas [1]. This criterion is based on exclusion of three entities that have been recognized as part of these subtypes: Primary Cutaneous CD4-Positive Small/Medium T-Cell Lymphoma (CD4+ SMTL), Primary Cutaneous CD8-Positive Aggressive Epidermotropic T-Cell Lymphoma (CD8+ AECTCL), and Primary Cutaneous Gamma/Delta T-Cell Lymphoma (CGD-TCL) [2, 3]. Each subtype has its own unique immunohistochemical and clinical characteristics [4]. Out of these subtypes, only CD4+ SMTL confers a good prognosis [2, 5]. CD8+ AECTCL usually presents with rapidly progressing necrotic nodules and plaques as in PTL-NOS, but with epidermotropic CD8+ atypical lymphocytes [4].

Due to the rarity of PTL-NOS, there are gaps in the knowledge about evidence-based treatments and survival [4]. However, it has been shown that age greater than sixty, Eastern Cooperative Oncology Group (ECOG) performance status of equal to or greater than two, lactate dehydrogenase levels at normal values or above, and involvement of the bone marrow are independent predictors of decreased survival [6].

Due to the rapidly evolving nature of PTL-NOS, treatment usually includes systemic chemotherapy and/or hematopoietic stem cell transplantation [2, 4]. Systemic chemotherapy often includes the CHOP multiagent regimen [4]. Since PTL-NOS presents in such an aggressive nature, most other treatments for CTCL such as interferon-alpha, retinoids, PUVA light therapy, and localized radiotherapy are bypassed [7]. Studies have shown that cytokine treatments such as interferon-alpha, which are useful in Mycosis Fungoides and Sezary Syndrome, not only are ineffective in PTL-NOS, but may also exacerbate the condition [8]. Even with systemic chemotherapy and stem cell transplantation, prognosis is unfortunately still very poor for patients with PTL-NOS [4].

## 4. Conclusion

CTCL should be suspected in patients with patches, plaques, erythroderma, or papules that persist or multiply despite conservative treatment. Initial biopsies are often nondiagnostic. Diagnosis requires a high degree of suspicion and multiple rebiopsies are often necessary to make the diagnosis. Although Mycosis Fungoides is the most common form of CD4-positive CTCL, it is prudent to consider PTL-NOS as the initial diagnosis for such a presentation, bearing in mind the rarity of the disease and nonspecific immunohistochemical analysis. One must have a high index of suspicion for alternative diagnoses if clinical progression is inconsistent with usual course of disease.
